# Dramatic increases in knowledge, attitudes and practices of COVID-19 observed among low-income households in the Philippines: A repeated cross-sectional study in 2020

**DOI:** 10.7189/jogh.12.05015

**Published:** 2022-05-21

**Authors:** Lincoln L Lau, Natalee Hung, Daryn J Go, Mia Choi, Warren Dodd, Xiaolin Wei

**Affiliations:** 1 International Care Ministries Inc., Manila, Philippines; 2 School of Public Health Sciences, University of Waterloo, Waterloo, Canada; 3 Dalla Lana School of Public Health, University of Toronto, Toronto, Canada

## Abstract

**Background:**

The COVID-19 pandemic has severely impacted populations globally, and knowledge, attitudes and practices (KAPs) surrounding the virus have necessarily evolved. This study was conducted in partnership with International Care Ministries (ICM), a Philippine-based non-governmental organization that runs the “Transform” poverty alleviation program. The main objective of this study was to describe the changes in COVID-19 KAPs among households experiencing extreme poverty in the Philippines over an 8-month period.

**Methods:**

A KAP questionnaire was integrated into the household survey collected as part of ICM’s regular monitoring and evaluation of “Transform”. Data collection for the first survey was conducted between February 20 and March 13, 2020, and the second survey was conducted between November 12 and December 12, 2020. Frequencies and proportions were calculated to describe the respondents’ responses and the Kruskal-Wallis test was used to assess if there were significant differences in KAP identification between the two time points.

**Results:**

We observed a distinct increase across all KAP domains. Over 90% of study participants were able to correctly identify COVID-19 transmission modes and preventive measures, and an even higher percentage reported adopting these measures. However, the intention to seek care from public hospitals for COVID-19 symptoms dropped from 43.6% to 28.4%, while reports of self-treatment using stored medicines or antibiotics increased. Trust in community members and local health authorities was correlated with higher knowledge and practice scores.

**Conclusions:**

Our study results reflect the extraordinary speed of information dissemination and behaviour change globally over the course of the pandemic, but they also highlight the changes in KAP that show the additional challenges faced by populations experiencing poverty in the Philippines. Prioritization of reducing inequities in the implementation and adoption of the evolving public health measures will be integral as the pandemic continues.

COVID-19 has spread wider and persisted for longer than most people expected, but the end of the pandemic is still far from sight. Virtually no country has been left untouched by COVID-19, with most having experienced multiple waves of infections and the unintended consequences of imposing a series of measures to control its spread [1]. In the Philippines, when community transmission of the virus was detected towards the end of February 2020 [2], the government rapidly imposed sweeping enhanced community quarantine (ECQ) measures, including strict stay-at-home orders, curfews, the suspension of public transport and any operation of economic activity that was deemed “non-essential” [3]. For many regions in the Philippines, especially those considered “high-risk”, these measures were in place for months, but the consequences of limiting economic activity led to the loosening of lockdown restrictions in May 2020 [4]. However, an accelerated spread of the virus soon followed, with a daily average of over 2000 incident cases of COVID-19 from July to September 2020, more than double the figures in June 2020 [2]. Hospitals, especially in the national capital region, came close to full capacity [5,6]. To ease the burdens on the health care system, ECQ measures were reimposed in August 2020, with modifications to allow some industries to operate at reduced capacities [4]. With a return to stay-at-home orders and restrictions on economic activity, life was significantly disrupted for most of the population in the Philippines, with magnified effects for those without fixed incomes or savings to sustain living costs [7].

A previous study on the knowledge, attitudes, and practices (KAP) of COVID-19 among households experiencing extreme poverty in the Philippines was conducted between February 20 and March 13, 2020 [8]. These participants were surveyed before the first surge in COVID-19 cases that overwhelmed the health care system and led to wide enforcement of lockdown measures. The survey was repeated at the end of 2020 and captured an entirely different landscape: while infection numbers had declined slightly from the summer to about 1000 cases daily, populations had lived through extended lockdown restrictions and ongoing community spread of the virus. Given the severe and widespread impacts of the pandemic, in addition to ongoing information campaigns about ways to combat COVID-19, improvements in knowledge surrounding the virus and adherence to preventive measures over time would be expected. However, there are vast differences in how diverse populations have experienced the pandemic, in which inequalities in socioeconomic resources can heavily influence access to information, protective equipment, and health care [6,9].

The objectives of this study were 3-fold: 1) To describe the changes in COVID-19 KAPs among households experiencing extreme poverty in the Philippines over a period of about 8 months, 2) to examine the socio-demographic characteristics correlated with COVID-19 knowledge and practices during the second survey period, and 3) to assess whether receiving targeted health education was correlated with greater KAP towards COVID-19.

## METHODS

### Study design

This study was conducted in partnership with International Care Ministries (ICM), a Philippine-based non-governmental organization (NGO) working to support extreme low-income households through a poverty-alleviation program called “Transform”. ICM’s “Transform” program works exclusively with individuals and households experiencing extreme poverty, defined by the organization as households that report having a daily income below US$0.50 per person [10]. Embedded within “Transform” are health training sessions that ordinarily focus on areas such as hygiene, sanitation, disease prevention and nutrition. As the pandemic progressed, a module on COVID-19 was integrated into the program in April 2020, and included topics such as transmission routes of COVID-19, social distancing, hand hygiene reminders, and encouragement of face mask use. Not all participants who were enrolled were able to complete the “Transform” program due to various disruptions related to COVID-19, however, this allowed us to examine the effect of health education on KAP in this context.

**Figure 1:**
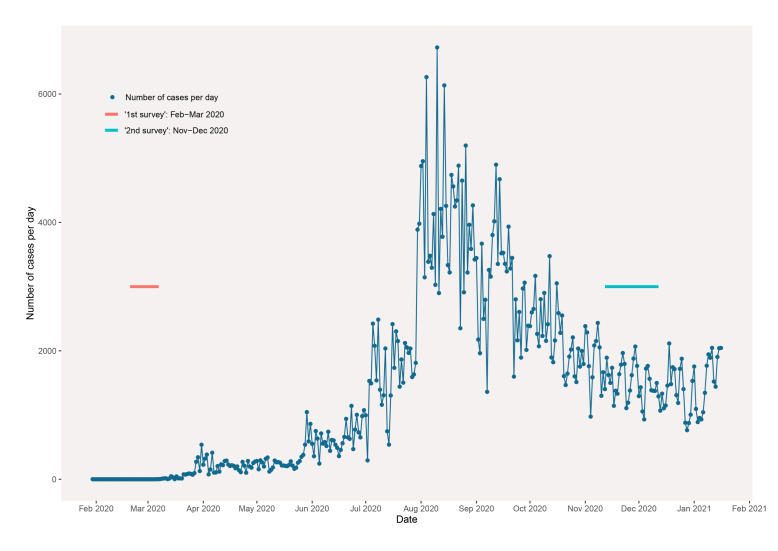
Confirmed COVID-19 cases over time in the Philippines and International Care Ministries (ICM) survey periods.

The survey was carried out from November 12, 2020, to December 12, 2020, about 8 months since the first COVID-19 KAP questionnaire was administered (**Figure 1**) [8]. As with the previous study, the household survey data were collected as part of ICM’s regular monitoring and evaluation of “Transform”. For study purposes, we will refer to the first survey as “the early pandemic period”, and the second survey as the “mid-pandemic period”, as the pandemic is ongoing and still evolving. All “Transform” participants were pre-screened by ICM and partnering community leaders to be experiencing extreme poverty based on self-reported income and physical dwelling characteristics. The subset of participants surveyed was different than in the previous study, but given Transform’s homogenous recruitment process, both samples should represent the same socio-demographic background.

The KAP questions for the respondents were identical to those used during the early pandemic period, covering the domains of knowledge (awareness of the COVID-19, modes of transmission, effective preventive measures), attitudes (perception of life disruption due to COVID-19, worry about contracting the virus), practices (preventive measures adopted), and sources consulted for information about COVID-19 (see **Online Supplementary Document** for the full list of questions). These surveys were translated from English to local languages (Tagalog, Bisaya, and Hiligaynon), and subsequently back-translated to ensure accuracy.

COVID-19 precautions were implemented throughout the data collection process to ensure the safety of the survey enumerators and the respondents. Enumerator training included modules on proper mask usage and hygiene practices, safety precautions, and surveying strategies, such as organizing seating arrangements to maintain social distancing and surveying outdoors or in well-ventilated areas. These training sessions were conducted online where possible, and arrangements for grouped off-site online training were made for enumerators who were unable to access the online training at home due to poor internet connectivity, in compliance with local gathering restrictions. Reverse transcription-polymerase chain reaction (RT-PCR) COVID-19 tests were provided at ICM’s regional field office sites, and enumerators were required to submit a negative test prior to entering the communities. Data collection periods were phased across field office sites to account for testing needs and local travel restrictions. Upon arrival at the household, enumerators were instructed to first inquire if anyone in the household was confirmed or suspected of being infected with COVID-19, being monitored for COVID-19, or currently undergoing quarantine. If so, this was noted by the enumerators and the survey was not conducted.

Households in rural, urban, and coastal settings from the following twenty provinces in the Philippines were surveyed: Palawan, North Cotabato, Zamboanga Del Sur, Misamis Occidental, Zamboanga Del Norte, Sultan Kudarat, Zamboanga Sibugay, Sarangani, Davao Del Sur, South Cotabato, Guimaras, Siquijor, Antique, Aklan, Capiz, Iloilo, Negros Oriental, Bohol, Negros Occidental, and Cebu.

### Statistical analysis

Frequencies and proportions were calculated for each KAP question from both surveys conducted during the early pandemic period and the mid-pandemic period. Given that our continuous outcome measures did not follow a normal distribution via examination by the Shapiro-Wilk test, the Kruskal-Wallis test was used to assess whether there were significant differences (*P* < 0.05) in KAP identification between the two time points.

Additional analysis was conducted on responses from the mid-pandemic period. To examine the socio-demographic characteristics correlated with COVID-19 knowledge and practices, the Kruskal-Wallis test was used. Summary scores (range = 0-5) for the number of COVID-19 transmission modes identified, the number of COVID-19 preventive measures identified, and the number of COVID-19 preventive measures taken were constructed as the outcome variables. The Kruskal-Wallis test indicated whether the means of the outcome variables were significantly different between the categories of each socio-demographic variable, which included: income level, geographical location, educational attainment, access to mobile phones or televisions, social ties with regional health and government units, and whether the respondent completed the “Transform” program.

The correlation of these socio-demographic characteristics with attitudes towards COVID-19 during the mid-pandemic period was also examined. Whether respondents were worried about contracting COVID-19 was used as the binary outcome measure. Odds ratios and 95% confidence intervals (CIs) were calculated using multivariable logistic regression. The number of COVID-19 transmission modes identified and whether respondents felt that their lives had been changed by the virus were also included as predictors in the model. All statistical analyses were conducted using Stata 13 (StataCorp, College Station, Texas, USA) [11] and R version 3.2.3 (R Core Team, R Foundation for Statistical Computing, Vienna, Austria) [12].

### Ethics approval

Data collection was approved by the University of Toronto Research Ethics Board (Protocol Number: 39138), and verbal informed consent for survey participation was obtained from all respondents.

## RESULTS

### Early pandemic period vs mid-pandemic period

#### Respondent characteristics

A total of 3354 participants representing 174 communities were targeted for the mid-pandemic period survey, of which 2453 participants from 158 communities were successfully surveyed (78.2%). This response rate was lower than for the early pandemic period survey (91.6%; 2224 participants) as some communities could not be accessed due to local COVID-19 travel restrictions. **Table 1** compares the background characteristics of survey respondents from both the early (t_1_) and mid-pandemic period (t_2_). The samples were highly comparable, as no significant differences were detected between them on most demographic characteristics: notably, over 90.0% of respondents at both time points were female, and the average age was 41.7 years old (SD = 14.4) in the mid-pandemic period, compared to 41.3 years old (SD = 14.6) in the earlier survey. From the characteristics where there were significant differences, we were able to observe some of the socio-economic ramifications due to COVID-19 in this population. Where 57.0% of respondents were living on less than US$1.00 a day in early 2020, this figure had risen by 65.0% by the end of the year. As most of the respondents are day labourers, farmers, fishermen, and under-employed, frequent work disruptions caused by anti-epidemic measures, such as regional lockdowns and community quarantine restrictions, can have tremendous impacts on their income sources. The apparent movement of respondents from urban to rural areas may also reflect the decisions of households to relocate away from cities with strict and prolonged implementation of lockdown restrictions.

**Table 1:** Characteristics of study respondents, comparison of samples surveyed during the early vs mid-pandemic period

Characteristic		Early pandemic period (n = 2224)		Mid-pandemic period (n = 2453)		*P*-value*
**n**	**%**	**n**	**%**
Sex	Female	2061	92.7	2238	91.2	0.072
	Male	163	7.3	215	8.8	
Age	Less than 20 y old	50	2.2	37	1.5	0.700
	20-39 y old	1080	48.6	1198	48.8	
	40-59 y old	783	35.2	882	36.0	
	60 y old and above	311	14.0	336	13.7	
Education	No education	48	2.2	73	3.0	0.026
	Elementary	929	41.8	1066	43.5	
	Partial high school	818	36.8	894	36.4	
	High school graduate or ALS	213	9.6	207	8.4	
	Partial college or vocational school	165	7.4	157	6.4	
	Completed college-level or higher	51	2.3	56	2.3	
Income per person per day (self-reported)	Less than $1.00	1262	56.7	1572	64.1	<0.001
	$1.00–$2.00	710	31.9	669	27.3	
	Greater than $2.00	248	11.2	186	7.6	
	Refuse to answer	0	0.0	26	1.1	
Number of mobile phones	None	491	22.1	584	23.8	0.062
	One	1064	47.8	1188	48.4	
	Two	480	21.6	482	19.6	
	Three or more	189	8.5	199	8.1	
Electricity	Yes	1773	79.7	1991	81.2	0.200
	No	451	20.3	462	18.8	
Television	Yes, and it is in working condition.	1084	48.7	1162	47.4	0.700
	Yes, but it is out of order or not working anymore	116	5.2	229	9.3	
	No	1024	46.0	1062	43.3	
Geotype	Coastal	283	12.7	317	12.9	0.047
	Rural	1725	77.6	1965	80.1	
	Urban	200	9.0	159	6.5	
	Refuse to answer	0	0.0	12	0.5	
PhilHealth	Yes	1391	62.5	1544	62.9	0.200
	No	824	37.1	856	34.9	
	I don't know	7	0.3	5	0.2	
	Refuse to answer	0	0.0	48	2.0	

ALS – alternative learning system

*Kruskal-Wallis rank sum test

#### Information sources

Traditional media were still the dominant sources of information about COVID-19 (**Table 2**). Television remained the most popular information source with about 85% (t_2_) of respondents reporting using it to stay up to date about the virus, similar to the early pandemic period survey’s report (*P* = 0.600). The next most frequently sought information source, the radio, saw a dramatic increase in its reported use from 56.1% (t_1_) to 74.2% (t_2_) (*P* < 0.001). Compared to the earlier survey, there were substantial increases in respondents who reported consulting other community members for information about COVID-19, notably friends, family or neighbours (t_1_ = 43.4%, t_2_ = 62.2%; *P* < 0.001) and local government officials (t_1_ = 22.9%, t_2_ = 54.7%; *P* < 0.001). While the use of social media (t_1_ = 20.7%, t_2_ = 33.9%; *P* < 0.001) and internet sources (t_1_ = 11.0%, t_2_ = 18.8%; *P* < 0.001) also increased, the use of digital media was comparatively less common.

**Table 2:** Information sources for COVID-19 reported by survey respondents, early vs mid-pandemic period

Question	Early pandemic period (n = 2090)		Mid-pandemic period (n = 2437)		*P* value*
**n**	**%**	**n**	**%**
**Where did you learn and stay up to date about COVID-19?**					
Television	1786	85.5	2070	84.9	0.600
Radio	1173	56.1	1808	74.2	<0.001
Social media (Facebook, Instagram, etc.)	432	20.7	825	33.9	<0.001
Internet (websites, blogs, etc.)	230	11.0	458	18.8	<0.001
Friends, relatives, and/or neighbours	908	43.4	1516	62.2	<0.001
Local government officials	479	22.9	1334	54.7	<0.001
Announcements at work	51	2.4	180	7.4	<0.001
Other	10	0.5	5	0.2	0.110

*Kruskal-Wallis rank sum test

#### COVID-19 knowledge, attitudes and practices

The mid-pandemic survey was conducted when COVID-19 was widespread both globally and in the Philippines. At this time, 99.3% of respondents reported having heard of the virus, compared to 94.0% (t_1_) during the early pandemic period (**Table 3**; *P* < 0.001). It is evident that the effects of the virus and the associated policy restrictions to curb its spread were far-reaching, as the percentage of those who reported having their lives disrupted by COVID-19 increased from 54.0% (t_1_) to 90.5% (t_2_) (**Table 4**; *P* < 0.001). This heightened awareness of the virus was also reflected in increased knowledge, as all COVID-19 transmission routes presented in the survey were identified by over 90% (t_2_) of respondents. Identification of relevant preventive practices was also largely accurate, with increases in the selection of handwashing (t_1_ = 82.2%, t_2_ = 95.2%; *P* < 0.001), face masks (t_1_ = 49.0%, t_2_ = 94.4%; *P* < 0.001), hand sanitizers (t_1_ = 44.4%, t_2_ = 81.2%; *P* < 0.001), and staying away from people who are sick (t_1_ = 32.4%, t_2_ = 81.1%; *P* < 0.001) as ways to protect themselves from COVID-19. An increase was also seen for the option of avoiding large crowds (t_1_ = 40.6%, t_2_ = 67.3%; *P* < 0.001), but its relatively lower identification as a preventive practice may be because it is less relevant in remote rural and coastal areas. Drastic improvements in practices were also observed compared to the early pandemic period, with almost universal implementation of recommended preventive measures among survey respondents, including avoiding crowded places (t_1_ = 62.9%, t_2_ = 96.9%; *P* < 0.001), hand washing (t_1_ = 89.9%, t_2_ = 98.9%; *P* < 0.001), having access to hand sanitisers (t_1_ = 71.0%, t_2_ = 96.0%; *P* < 0.001), and wearing face masks (t_1_ = 28.0%, t_2_ = 98.2%; *P* < 0.001). Notably, in both surveys, the percentage of respondents who reported *practising* these preventive measures was greater than the percentage of those who *identified* it as a preventive measure.

**Table 3:** Knowledge about COVID-19 among income-poor households in the Philippines, early vs mid-pandemic period

Questions	Early pandemic period (n = 2090)		Mid-pandemic period (n = 2437)		*P*-value†
**n**	**%**	**n**	**%**
**Knowledge**					
Have you heard of COVID-19?*					<0.001
Yes	2090	94.0	2437	99.3	
No	117	5.3	15	0.6	
Don't know	17	0.8	1	<0.1	
***Can COVID-19 be transmitted (caught or spread) by…***					
*Coughing and sneezing?*					<0.001
Yes	1870	89.5	2319	95.2	
No	116	5.6	79	3.2	
Don't know	104	5.0	39	1.6	
*Face to face talking?*					<0.001
Yes	1735	83.0	2299	94.3	
No	206	9.9	90	3.7	
Don't know	149	7.1	48	2.0	
*Handshakes or hugs?*					<0.001
Yes	1698	81.2	2289	93.9	
No	252	12.1	102	4.2	
Don't know	140	6.7	46	1.9	
*Touching an item someone else touched?*					<0.001
Yes	1518	72.6	2242	92.0	
No	376	18.0	143	5.9	
Don't know	196	9.4	52	2.1	
*Sharing and eating from the same dish?*					<0.001
Yes	1774	84.9	2294	94.1	
No	193	9.2	99	4.1	
Don't know	123	5.9	44	1.8	
***How can you protect yourself against COVID-19?***					
Hand washing	1719	82.2	2321	95.2	<0.001
Face masks	1024	49.0	2301	94.4	<0.001
Hand sanitizer	927	44.4	1980	81.2	<0.001
Staying away from people who are sick	677	32.4	1977	81.1	<0.001
Vitamins, Calamansi tea or other citrus fruit, herbal remedies	683	32.7	1218	50.0	<0.001
Avoiding large crowds	849	40.6	1641	67.3	<0.001
Drinking alcohol	66	3.2	103	4.2	0.059
Changing clothes often or after being in public	225	10.8	691	28.4	<0.001
Ginger pouch	24	1.1	186	7.6	<0.001
Other	72	3.4	27	1.1	<0.001
***If you have symptoms like fever, cough, and sore through, what would you do? (select all that apply)***					
Stay at home and wait to get better	762	36.5	1610	66.1	<0.001
Use stored medicine at home	724	34.6	1226	50.3	<0.001
Contact *barangay* health worker (community health worker)	1033	49.4	1332	54.7	<0.001
Seek antibiotics	423	20.2	693	28.4	<0.001
Visit RHU	720	34.4	948	38.9	0.002
Visit a pharmacy	309	14.8	480	19.7	<0.001
Visit a public hospital	911	43.6	692	28.4	<0.001
Visit a private hospital	252	12.1	275	11.3	0.400
See the local hilot (traditional medicine)	145	6.9	323	13.3	<0.001
Other	46	2.2	70	2.9	0.200
I don't know	15	0.7	7	0.3	0.038

RHU – rural health unit

*The sample size for this question was n = 2224 and n = 2453 for the early pandemic period and the mid-pandemic period, respectively. Only individuals who responded “Yes” were asked to complete the subsequent knowledge, attitudes, and practices (KAP) questions.

†Kruskal-Wallis rank sum test

**Table 4:** Attitudes and practices toward COVID-19 among income-poor households in the Philippines, early vs mid-pandemic period

Questions	Early pandemic period (n = 2090)		Mid-pandemic period (n = 2437)		*P*-value*
**n**	**%**	**n**	**%**
**Attitudes**					
***Has your daily life been disturbed (interrupted, changed) by COVID-19?***					<0.001
Yes	1128	54.0	2205	90.5	
No	962	46.0	232	9.5	
***Do you worry about contracting COVID-19?***					<0.001
Yes	1679	80.3	2320	95.2	
No	411	19.7	117	4.8	
**Practices**					
***Because of COVID-19…***					
*Do you avoid crowded places?*					<0.001
Yes	1314	62.9	2361	96.9	
No	752	36.0	73	3.0	
Don't know	24	1.1	3	0.1	
*Do you wash your hands more frequently?*					<0.001
Yes	1879	89.9	2410	98.9	
No	196	9.4	21	0.9	
Don't know	15	0.7	6	0.2	
*Do you have access (can you buy or receive) to alcohol, hand sanitizer? (or have you bought more recently?)*					<0.001
Yes	1484	71.0	2340	96.0	
No	577	27.6	90	3.7	
Don't know	29	1.4	7	0.3	
*Do you wear a face mask now because of the new coronavirus?*					<0.001
Yes	585	28.0	2394	98.2	
No	1479	70.8	40	1.6	
Don't know	26	1.2	3	0.1	
*Do you keep a distance from people with influenza-like symptoms (flu/colds)?*					<0.001
Yes	1378	65.9	2113	86.7	
No	696	33.3	320	13.1	
Don't know	16	0.8	4	0.2	

*Kruskal-Wallis rank sum test

There were also changes in what respondents reported they would do if they displayed COVID-19 symptoms. There were noteworthy increases in the number of people who reported they would stay at home and wait to get better (t_1_ = 36.5%, t_2_ = 66.1%; *P* < 0.001), use stored medicine at home (t_1_ = 34.6%, t_2_ = 50.3%; *P* < 0.001), or seek antibiotics (t_1_ = 20.2%, t_2_ = 28.4%; *P* < 0.001), whereas the intention to visit public hospitals saw a substantial drop from 43.6% (t_1_) to 28.4% (t_2_) (*P* < 0.001).

### Determinants of COVID-19 KAPs during the mid-pandemic period

We further examined the socio-demographic characteristics correlated with COVID-19 knowledge and practices during the mid-pandemic period (**Table 5**). In general, those with higher educational attainment identified and practised a greater number of preventive measures against COVID-19. Having access to at least one phone or a working television was also correlated with adopting preventive practices. Trust in the community, especially in local health clinics (ie, *barangay* health stations or rural health units [RHUs]), possibly played a key role in determining COVID-19 knowledge and practices. Respondents who reported higher levels of trust in their neighbours, on average, correctly identified and adopted more COVID-19 preventive measures. Higher levels of trust in respondents’ barangay health station or RHU were correlated with greater average scores on all three outcomes: correct identification of COVID-19 transmission routes, and preventive measures identified and taken. Lastly, and perhaps surprisingly, being a graduate of “Transform” did not have a significant effect on KAP scores.

**Table 5:** Groupwise means and results of Kruskal-Wallis Test on demographic and social capital determinants of COVID-19 knowledge and practices, mid-pandemic period (n = 2345)

Variables	Number of COVID-19 transmission modes identified				Number of COVID-19 preventive measures identified				Number of COVID-19 preventive measures taken			
**Mean**	**SD**	**χ^2^**	***P*-value**	**Mean**	**SD**	**χ^2^**	***P*-value**	**Mean**	**SD**	**χ^2^**	***P*-value**
**Income**			5.33	0.070			2.96	0.228			0.25	0.884
Less than $1.00	4.71	0.92			4.18	1.07			4.77	0.53		
$1.00-$2.00	4.76	0.89			4.19	1.04			4.75	0.62		
Greater than $2.00	4.73	1.03			4.32	1.00			4.74	0.66		
**Geotype**			1.99	0.370			2.54	0.281			4.45	0.108
Urban	4.52	1.30			4.27	0.94			4.72	0.61		
Rural	4.74	0.87			4.17	1.08			4.76	0.58		
Coastal	4.73	0.98			4.30	0.96			4.84	0.41		
**Education**			5.72	0.334			15.14	0.010			24.95	0.000
No education	4.68	1.07			4.22	1.13			4.73	0.69		
Elementary	4.71	0.93			4.12	1.10			4.71	0.65		
Partial high school	4.73	0.91			4.20	1.06			4.82	0.46		
High school graduate or ALS	4.71	0.93			4.38	0.89			4.81	0.48		
Partial college or vocational school	4.69	0.97			4.39	0.87			4.80	0.55		
Completed college-level or higher	4.91	0.68			4.35	0.89			4.91	0.29		
**Has at least one phone**			1.26	0.262			1.45	0.229			11.97	0.001
No	4.68	0.98			4.15	1.09			4.70	0.65		
Yes	4.73	0.91			4.21	1.05			4.79	0.53		
**Has a working TV**			2.26	0.133			0.94	0.332				
No	4.71	0.92			4.21	1.06			4.74	0.59	6.56	0.010
Yes	4.73	0.93			4.18	1.05			4.79	0.54		
**Trusts neighbor**			6.65	0.156			47.36	<0.001			13.12	0.011
No trust	4.68	0.97			4.38	0.90			4.77	0.50		
Tentatively trust	4.79	0.85			4.43	0.94			4.84	0.43		
Neutral	4.72	0.91			4.11	1.03			4.79	0.48		
Moderately trust	4.71	0.94			4.11	1.10			4.71	0.67		
Very trusting	4.68	0.98			4.30	1.09			4.78	0.56		
**Trusts *barangay* health station or RHU**			15.17	0.004			11.18	0.025			17.78	0.001
No trust	4.08	1.78			3.58	1.38			4.58	0.51		
Tentatively trust	4.72	0.98			4.17	1.08			4.79	0.56		
Neutral	4.59	1.12			4.13	1.06			4.73	0.63		
Moderately trust	4.78	0.81			4.19	1.07			4.75	0.56		
Very trusting	4.76	0.84			4.28	1.01			4.83	0.49		
**Knows the *barangay* captain**			3.38	0.066			0.24	0.624			4.85	0.028
No	4.91	0.41			4.26	0.99			4.70	0.50		
Yes	4.71	0.94			4.19	1.06			4.77	0.57		
**“Transform” graduate**			2.63	0.105			0.40	0.525			0.05	0.817
No	4.70	0.95			4.17	1.09			4.75	0.63		
Yes	4.73	0.92			4.21	1.03			4.78	0.51		

SD – standard deviation, RHU – rural health unit, ALS – alternative learning system

In the investigation of risk perception attitudes (**Table 6**), similar to the early pandemic period, respondents who had greater knowledge of transmission modes (OR = 1.67; 95% CI = 1.46-1.90) and those who reported having their daily life disturbed by COVID-19 (OR = 6.04; 95% CI = 3.66-9.96) were more likely to report being worried about contracting the virus.

**Table 6:** Results of logistic regression of demographic, social capital and KAP determinants of COVID-19 risk perception attitudes, mid-pandemic period (n = 2345)

Variables	Worried about contracting the virus	
**OR (95% CI)**	***P*-value**
**Income**		
Less than $1.00	(ref)	
$1.00-$2.00	0.99 (0.54-1.79)	0.967
Greater than $2.00	0.56 (0.25-1.26)	0.158
**Geotype**		
Rural	(ref)	
Urban	6.13 (0.90-41.76)	0.064
Coastal	0.87 (0.42-1.81)	0.720
**Has at least one phone**	0.56 (0.29-1.07)	0.080
**Has a working TV**	1.35 (0.79-2.30)	0.274
**Trusts *barangay* health station or RHU**		
No trust	(ref)	
Tentatively trust	2.33 (0.35-15.47)	0.380
Neutral	2.91 (0.49-17.32)	0.240
Moderately trust	4.44 (0.74-26.58)	0.103
Very trusting	3.15 (0.52-19.05)	0.212
**Knows the *barangay* captain**	0.32 (0.03-3.63)	0.359
**“Transform” graduate**	1.20 (0.72-1.99)	0.492
**Life has been disturbed (interrupted, changed) by the new coronavirus**		
No	(ref)	
Yes	6.04 (3.66-9.96)	<0.001
**Number of COVID-19 transmission modes identified**	1.67 (1.46-1.90)	<0.001

OR – odds ratio, RHU – rural health unit

## DISCUSSION

The COVID-19 pandemic has severely impacted populations globally, and capturing the experiences of those in lower socioeconomic positions is crucial to supporting the ongoing development of equitable pandemic management strategies [13]. In this study, we had the opportunity to examine changes in COVID-19 KAPs among households experiencing extreme poverty in the Philippines over a period of about 8 months.

### Changes in KAP over time

During the early pandemic period, we found that knowledge of transmission routes was high, but there was low identification and adoption of appropriate preventive measures against COVID-19 [8]. By the mid-pandemic period, there was a dramatic increase across all KAP domains: over 90% of our study respondents correctly identified COVID-19 transmission modes and preventive measures, with an even higher percentage reporting adopting these measures. Due to the unique nature of this pandemic, these relatively rapid changes were not unexpected. With the speed and scale of the pandemic’s spread and the drastic mitigation measures implemented globally, remaining insulated from the effects of the virus, either directly via infection or indirectly as a result of changes to daily routines, was almost impossible. This reality likely fueled the sense of urgency to stay informed and respond appropriately, along with widespread fear and confusion, ceaseless supply of information, and the many lingering unknowns [14,15].

The changes in KAP responses were particularly apparent in the domain of implementation of preventive practices, where all measures except the keeping of distance from people with influenza-like symptoms were put into practice by over 95% of the respondents. Use of face masks saw the most dramatic increase, from 28.0% of respondents during the early pandemic period, to 98.2% during the mid-pandemic period, which likely reflected the massive shift in global recognition of face masking as an effective preventive measure [16-19]. Even with the expansion of face masks usage globally, cross-national comparisons show that adoption of this preventive measure in the Philippines was particularly high. A survey conducted in October 2020 found that, among the 27 countries surveyed, the Philippines was consistently the frontrunner in reports of face mask use outside the home, at work, in shops and on public transport [20]. Similar trends were observed for numerous other non-pharmaceutical interventions (NPIs) as well, where the Philippines reported one of, if not the highest, implementation of preventive practices such as improving hand sanitation and social distancing [21].

While the value placed on adhering to such NPIs has been generally higher among Asian countries compared to Europe and the Americas [22], these attitudes seem to be additionally magnified in the Philippines. Information campaigns organised by the Philippines’ Department of Health that promoted practices such as face masking, hand sanitation, and social distancing as the “minimum public health standards” against COVID-19 may be one reason for high NPI adherence [23]. However, increased frequency and consistency in public health messaging are unlikely to be the only reasons, as we also observed that some respondents who reported practicing certain measures had not identified them as preventive practices. In the Philippines, the government adopted a highly militarized approach in response to COVID-19, where the police and military were brought in to strictly enforce lockdown measures as early as March 2020 [4]. The rhetoric pushed by government officials was that the national action plan against COVID-19 is fundamentally rooted in discipline and compliance to the minimum public health standards, over and above testing, tracing, or scaling up the health care system [4]. The public was warned that violators of these standards or of community quarantine orders would be arrested, and these measures were stringently carried out, often without warning [24]. High implementation of preventive practices, therefore, may have been more in response to the government’s rhetoric of discipline and following orders, rather than the association of these practices as acts that reduce the risk of COVID-19 transmission.

Government and health authorities may have perceived the disciplinary approach in enforcement of pandemic response measures as necessarily strong due to the lack of health sector preparedness to respond. The primary care system in the Philippines was quickly overwhelmed with severe shortages in testing capacity, hospital beds, and medical workers [25,26], even though only those with serious or critical symptoms received referrals to public hospitals [27]. Due to the highly decentralized nature of the health system in the Philippines, health service quality and capacity differs across the country, with ensuing implications on primary care access for individuals experiencing poverty [28]. Our results are therefore not surprising, as they show that, during the mid-pandemic period, only 28.4% participants had the intention to seek treatment from public hospitals, compared to 43.6% during the early pandemic period. Concerns about visiting places with increased risk of disease transmission or mistrust surrounding infection prevention and control protocols may have decreased intentions to access and use public hospitals. Given the severely reduced income due to COVID-19 movement restrictions, many individuals experiencing extreme poverty would also not have been able to afford travel expenses to the health facility or any out-of-pocket expenses not covered by the national health insurance program [29]. The social costs of seeking care would have also had to be taken into consideration, including experiencing socioeconomic status discrimination at public health care facilities [29] or exclusion from their community due to the stigma associated with contracting the virus [30]. Health systems worldwide, even those that were thought to be prepared [31,32], have come under pressure as a result of COVID-19, so it is perhaps unsurprising that existing patterns of exclusion remained unchanged. Challenges to health care access are still magnified for those with limited resources, and the decision of our participants *not* to seek care from public institutions during times of crises speaks volumes about the long-standing inequities in the organisation of the Philippines’ health system.

We also observed a rise in reports of self-treatment using stored medicine at home, which would have likely included over-the-counter medication for relief of respiratory symptoms associated with COVID-19. However, with the volume of information sharing and the potential for misinformation, the increase might have also reflected the increased use of drugs that have limited or inconclusive evidence as a treatment against the virus. The trend of turning to antibiotics for treatment has similar implications. Antibiotics are not effective against viral diseases such as COVID-19 and are only appropriate if patients have a bacterial co-infection, and their misuse can contribute to the threat of antimicrobial resistance [33]. With public health care systems at full capacity, people experiencing poverty face even higher barriers to health care access and are at risk of being pushed into seeking alternative means of self-treatment that may be misinformed.

### The role of trust in public health messaging

Given the remarkable increases in KAP scores between the two data collection periods, further analyses using only mid-pandemic data were conducted for additional insights into the factors that were beneficial for improving KAP in this population. Interestingly, KAP scores did not differ significantly between ICM’s “Transform” graduates who had received targeted health training about COVID-19 and those who had not completed the training. We may attribute the apparent lack of effect of targeted health education to the unique nature of COVID-19; with its far-reaching effects, respondents were likely saturated with information from other channels about transmission modes, preventive practices, and the importance of implementing them [34]. Conversely, it was trust in neighbours and local health clinics that was correlated with higher KAP scores, and this effect was still detected despite many participants attaining near-perfect scores. In a large-scale public health crisis, while health education and public health messaging are exceedingly important, it appears that trust will help the messaging to be *accepted* [35,36]. The punitive rhetoric in the Philippines, however, seems to be cultivating the opposite sentiment, with public surveys reporting decreasing satisfaction in the government’s handling of the pandemic and confidence in health authorities [37,38]. While the militaristic and police-centric approach may have been effective in achieving high adoption of preventive practices in the short term, the long-term effects of such an approach that negates the importance of trust, on both the institutional and interpersonal level, are unknown. There is mounting evidence that trust is a crucial factor that predicts willingness to cooperate with behavioural guidelines to curb the spread of COVID-19, not only in government authorities, but in fellow citizens and science as well [39]. The role of trust in strengthening the acceptance of public health communication will be important not only as we move into the next stages of the pandemic, but also for policies surrounding other infectious or non-communicable diseases.

### Strengths and limitations

The merit of this study is in its utilization of a repeated cross-sectional design, allowing for observation of how *timing* of data collection can drastically change KAP responses towards a disease, especially given COVID-19’s speed and scale of spread, but there are also limitations that must be acknowledged. There may be the potential of selection bias in the study sample. During data collection, the survey enumerators were instructed not to survey households with confirmed or suspected cases of COVID-19, or households in which there was someone currently undergoing quarantine. However, this only accounted for 0.3% of all households in the initial sampling frame sought, so we do not anticipate this exclusion criterion substantially altered the study results. With regards to health training within “Transform” and its effect on participants’ KAP scores, as the variable compared participants who had graduated from “Transform” (and therefore received the health training module on COVID-19) with those who had dropped out of the program before the training, we did not have a strict control group and were unable to make causal inferences. Also, as the survey results were based on self-reported rather than observed practices, some measures may have been overestimated due to social desirability bias. While we did not control for the possible effects of survey enumerators on responses, they had received training not to influence responses among participants.

Given the rapidly changing landscape of COVID-19, survey tools should also be updated as appropriate to capture the current state of knowledge and priorities. Future KAP studies, for example, should incorporate additional items to assess testing and vaccination. Additionally, as this study has demonstrated, KAP is time-sensitive, so it is imperative that studies report the time frame of data collection for meaningful interpretation of the results. This is likewise applicable to systematic reviews and meta-analyses of KAP studies, where the pooling of estimates without considering their position in the pandemic period should be avoided. As a complement to KAP studies, conducting qualitative research into determinants of trust in COVID-19 public health messaging and response measures would also be an important step to strengthen health service provision and access for populations experiencing poverty.

## CONCLUSIONS

The across-the-board increases in KAP towards COVID-19 observed in our study, alongside similar observations across multiple contexts and socioeconomic circumstances [21], reflect the extraordinary speed of information dissemination and behaviour change on a global scale over the course of the pandemic. While such trends are encouraging, it is important to bear in mind that the pandemic is rapidly evolving, and that the KAP items captured in our survey are only the most basic practices which are not comprehensive for long-term protection against the virus. With growing recognition that the pandemic will not simply end, there are several other measures being promoted in the transition to a “new normal”, such as regular testing and vaccinations [40]. There is a need for continued conversations about inequities in the implementation and adoption of these measures. Due to the social and economic repercussions associated with the uptake of these measures, such as the out-of-pocket financial costs of testing [41], facing social stigma and exclusion if the test returns positive [42], and rampant misinformation surrounding the safety of vaccination, we may expect to see divergences in KAP among populations experiencing poverty going forward. We cannot assume that there will be a similar alignment in KAP towards other public health measures, as we had observed in this study. To help ensure that the KAP of this population will be responsive to the changing circumstances surrounding COVID-19 as the pandemic progresses, fostering trust in community members, health authorities and governments will be more important than ever.

## Additional material


Online Supplementary Document
