# Supplementary material for: Dramatic increases in knowledge, attitudes and practices of COVID-19 observed among low-income households in the Philippines: A repeated cross-sectional study in 2020
Source: J Glob Health. 2022 May 21;12:05015. doi: 10.7189/jogh.12.05015 (PMC9123942; doi:10.7189/jogh.12.05015)
Supplement: Online Supplementary Document [file jogh-12-05015-s001.pdf]

Online supplementary material

Knowledge, attitudes and practices of COVID-19 among income-poor households in the Philippines: A repeated cross-sectional study

1 ICM 2019 Survey Covid-19 Questions

kn_0 Have you heard of the new coronavirus (Covid-19)?
1 Yes
0 No
-98 I don't know

kn_1 What do you think? Can the new coronavirus be transmitted (caught or spread) by…?:

kn_1a Cough and sneeze
1 Yes
0 No
-98 I don't know

kn_1b Face to face talking
1 Yes
0 No
-98 I don't know

kn_1c Handshakes or hugs
1 Yes
0 No
-98 I don't know

kn_1d Indirect hand contact (touching an item someone else touched)
1 Yes
0 No
-98 I don't know

kn_1e Food (sharing and eating from the same dish)
1 Yes
0 No
-98 I don't know

kn_2 If you have symptoms like fever, cough, and sore throat, what would you do?
Select all that are appropriate.
1 Stay at home and wait to get better
2 Use stored medicine at home
3 Contact *barangay* health worker
4 Seek antibiotics

5 Visit rural health unit (RHU)
6 Visit a pharmacy
7 Visit public hospital
8 Visit private hospital
9 Local hilot (traditional medicine)
-66 Other *(please specify)*
-98 I don't know

att_1 Has your daily life been disturbed (interrupted, changed) by the new coronavirus?
Has anything changed in your life since hearing about the new coronavirus?
1 Yes
1 No

att_2 Do you worry about contracting the new coronavirus?
1 No
2 Yes, a little
3 Yes, very much

pr_0 Since the news of the coronavirus…

pr_1 Do you avoid crowded places?
1 Yes
0 No
-98 I don't know

pr_2 Do you wash your hands more frequently?
1 Yes
0 No
-98 I don't know

pr_3 Do you have access (can you buy or receive) to alcohol, hand sanitizer? (or have you bought more recently?)
1 Yes
0 No
-98 I don't know

pr_4 Do you wear a face mask now because of the new coronavirus?
1 Yes
0 No
-98 I don't know

pr_4a (IF pr_4 = YES) What type of mask(s) do you wear?
Select all that are appropriate.
1 Motorcycle mask
2 Bandana
3 Surgical

4 N95
-66 Other (please specify)

pr_4b (IF pr_4 = NO) If no, is it because…
Select all that are appropriate.
1 All are out of stock
2 They are too expensive
3 I gave it to someone in my family
4 I don't think it helps against coronavirus
-66 Other *(please specify)*

pr_5 Do you keep a distance from people with influenza-like symptoms (flu/colds)?
Ex: people coughing, sneezing, with fever, with runny nose, etc.
1 Yes
0 No
-98 I don't know

inf_1 Where did you learn and stay up to date about the new coronavirus?
Select all that are appropriate.
2 Television (news)
3 Radio
4 Social media (Facebook, Instagram, etc.)
5 Internet (websites, blogs, etc.)
6 Friends, relatives, and/or neighbours
7 Government officials (local government officials, *barangay* health workers, etc.)
8 Announcements at work
-66 Other *(please specify)*

kn_3 How can you protect yourself against the new coronavirus?
Select all that are appropriate.
1 Hand washing
2 Face masks
3 Hand sanitizer
4 Social distancing (staying away from people who are sick)
5 Vitamins, Calamansi tea or other citrus fruit, herbal remedies
6 Avoiding large crowds
7 Drinking alcohol
8 Changing clothes often or after being in public
9 Ginger pouch
-66 Other *(please specify)*
